# The efficacy and safety of intra-articular platelet-rich plasma versus sodium hyaluronate for the treatment of osteoarthritis: Meta-analysis

**DOI:** 10.1371/journal.pone.0314878

**Published:** 2025-03-21

**Authors:** Qinglin Liu, Haijiao Ye, Yang Yang, Hao Chen

**Affiliations:** 1 Chongming Hospital Affiliated to Shanghai University of Medicine and Health Sciences, Shanghai, China; 2 Department of general Surgery, Chongming Hospital Affiliated to Shanghai University of Medicine and Health Sciences, Shanghai, China; 3 Department of Orthopaedics, Chongming Hospital Affiliated to Shanghai University of Medicine and Health Sciences, Shanghai, China; Government Law College, INDIA

## Abstract

**Background:**

Knee osteoarthritis (KOA) is a common degenerative joint disease that primarily affects the elderly individuals. Traditional treatments include medications and physical therapy, but recent attention has turned to platelet-rich plasma (PRP) and hyaluronic acid (HA) injection therapies.

**Objective:**

This meta-analysis aimed to evaluate the efficacy and safety of PRP combined with HA versus PRP alone in the treatment of KOA.

**Methods:**

We conducted a comprehensive literature search of the PubMed, Embase, and Cochrane Library databases, which included covering publications from their inception to July 2024. Studies comparing PRP+HA with PRP alone were selected. Data on visual analog scale (VAS) scores, WOMAC total scores, Lequesne scores, and adverse events were extracted. Statistical analysis was performed via Review Manager 5.3.5.

**Result:**

This meta-analysis included 16 studies involving a total of 1,384 patients. The VAS score comparison indicated that, in the long term, PRP combined with HA was more effective in reducing knee pain than PRP alone was (SMD: -0.30, 95% CI: -0.53 to -0.06, P =  0.01). The combined PRP and HA treatment achieved better results in terms of the WOMAC total score (MD =  -6.58, 95% CI: -10.65 to -2.52, P <  0.001). At the 6-month follow-up, the Lequesne index score comparison revealed that PRP combined with HA significantly improved knee pain scores compared with PRP alone (MD =  -1.38, 95% CI: -1.91 to -0.86, P <  0.001). In terms of adverse events, PRP+HA was associated with a lower risk of adverse events than PRP alone was (OR =  0.54, 95% CI: 0.33 to 0.85, P =  0.009).

**Conclusion:**

PRP combined with HA offers significant long-term benefits in pain relief and functional improvement over PRP alone for knee osteoarthritis, with better safety. The sequence of injection may influence treatment outcomes.

**Systematic review registration:**

PROSPERO CRD42024598691

## 1 Introduction

Knee osteoarthritis (KOA) is a prevalent joint disease, particularly among the elderly population. It is characterized by degenerative changes in the articular cartilage, osteophyte formation, and inflammatory responses in the surrounding tissues [1]. The primary symptoms of KOA include joint pain, stiffness, swelling, and a reduced range of motion. These symptoms not only severely impact patients’ quality of life but can also lead to functional disability and a diminished ability to perform daily activities.

Treatment options for KOA are diverse and include medications, physical therapy, intra-articular injections, and surgical interventions. Medications such as non-steroidal anti-inflammatory drugs (NSAIDs), analgesics, and disease-modifying anti-rheumatic drugs are commonly used to alleviate pain and control inflammation [2,3]. Physical therapy includes rehabilitation exercises, joint traction, and ultrasound treatment aimed at improving joint function and relieving symptoms [4]. However, long-term use of traditional medications and physical therapy may lead to issues such as reduced efficacy or side effects.

In recent years, intra-articular injection therapies have become an important treatment options for KOA. Two primary intra-articular injection therapies are platelet-rich plasma (PRP) and hyaluronic acid (HA) [5–7].

PRP is a biological product derived from the patient’s own blood, enriched with platelets and growth factors [8]. PRP injections promote the repair and regeneration of intra-articular soft tissues by releasing growth factors, potentially leading to significant pain relief and functional improvement [9]. PRP therapy has been used in various fields, including sports medicine and orthopedics, and has shown efficacy in the treatment of KOA [10].

HA is a naturally occurring polysaccharide with excellent lubricating and moisturizing properties [11]. HA injections can increase the viscosity of the joint fluid, improve joint lubrication, reduce pain, and help slow the progression of joint inflammation [12]. HA has a long history of clinical application and has been proven effective for some patients with KOA.

Despite the demonstrated efficacy of PRP and HA in treating knee osteoarthritis, the existing research results are not entirely consistent. Some studies suggest that PRP may outperform HA in terms of symptom and functional improvement, whereas others have not confirmed significant differences. Additionally, variations in PRP preparation methods, injection protocols, and patient differences may influence the assessment of efficacy. Although PRP and HA are generally considered relatively safe treatments, it is essential to systematically evaluate their potential adverse events. This study aimed to investigate the efficacy and safety of intra-articular injection of PRP combined with HA compared with PRP alone, providing evidence-based strategies for the treatment of KOA.

## 2 Methods

Our study is based on the Preferred Reporting Items for Systematic Reviews and Meta-Analyses (PRISMA) guidelines [13] and The review proposal was registered with PROSPERO: CRD42024598691.

### 2.1 Data sources

Two independent reviewers conducted a systematic search across multiple databases, including PubMed, Embase, and Cochrane Library. The search period was extended from the inception of each database up to July 2024. The search terms used included “PRP,” “platelet-rich plasma,” “hyaluronic acid,” “knee osteoarthritis,” and “injection.”

The inclusion criteria were as follows: (1) the participants were diagnosed with KOA. (2) The study compares intra-articular injection of PRP combined with HA in the experimental group with intra-articular injection of PRP alone in the control group. (3) Outcome measures include pain scores (e.g., VAS), functional scores (e.g., WOMAC), and adverse events. (4)The language of publication was English.

The exclusion criteria were as follows: (1) participants who were not diagnosed with KOA. (2) The study lacked essential clinical outcome data.

### 2.2 Data extraction

Two independent reviewers extracted relevant data from the articles via an Excel spreadsheet. The extracted information included basic details such as the study authors, publication year, country, study design, sex, sample size, age, injection method, dosage, frequency, and follow-up duration. The outcome measures included the Visual Analog Scale (VAS) scores, Western Ontario and McMaster Universities Osteoarthritis Index (WOMAC) total scores, Lequesne scores, and the incidence of adverse events.

### 2.3 Risk of bias assessment

In pooled clinical trials (PCTs), the Cochrane risk of bias tool is used to assess the risk of bias. This tool evaluates several aspects of the study: selection bias (e.g., the implementation of randomization and allocation concealment), performance bias (e.g., whether blinding was used for participants and researchers), detection bias (e.g., whether outcome assessment was blinded), attrition bias (e.g., handling of missing data and follow-up), and reporting bias (e.g., whether all prespecified outcomes were reported).

### 2.4 Statistical analysis

Statistical analyses were performed via Review Manager 5.3.5 software (Cochrane Collaboration, Oxford, UK). For continuous variables, standardized mean differences (SMDs) with 95% confidence intervals (95% CIs) were used, whereas for binary variables, odds ratios (ORs) and their 95% CIs were calculated. Additionally, heterogeneity was assessed using a threshold of P > 0.05 and I^2^ < 0%. When both conditions were met, indicating relatively uniform effect sizes, a fixed-effects model was used for the meta-analysis. If heterogeneity was present, suggesting a lack of uniformity in effect sizes, a random-effects model was applied. Subgroup analyses were conducted for outcomes with significant heterogeneity. To quantitatively assess the presence of publication bias for different outcome measures, both the leave-one-out sensitivity analysis and funnel plots were used for validation.

## 3 Results

### 3.1 Literature search and selection

**Fig 1:**
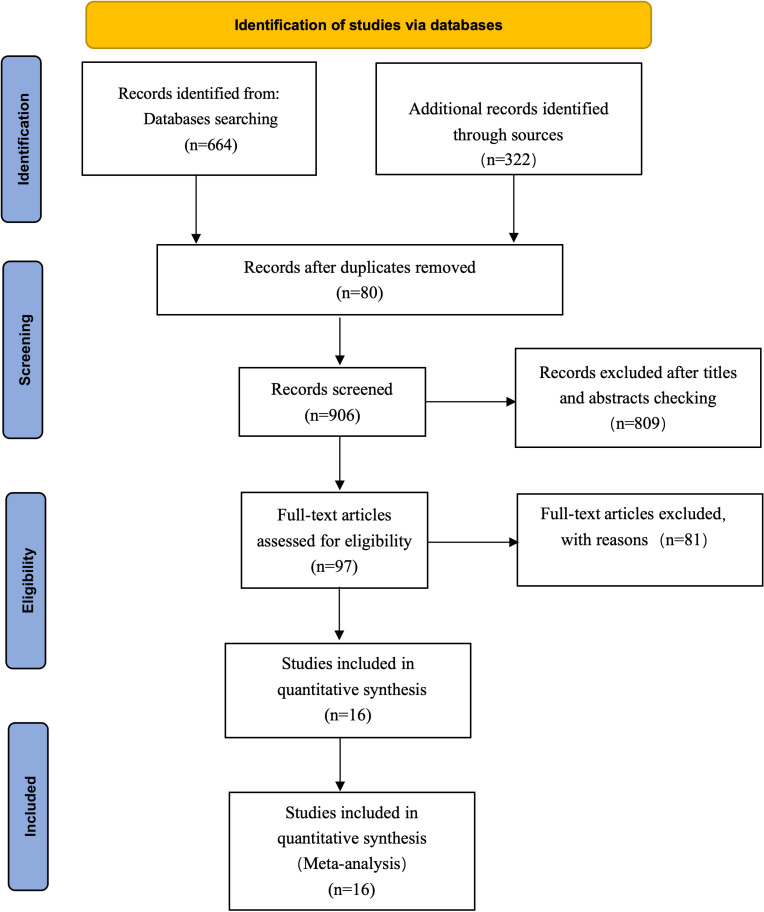
Constructing a flowchart for the included studies according to PRISMA Steps.

Initially, 986 articles related to the research question were identified. After excluding 80 duplicate articles were excluded, 97 articles were selected for full-text review based on titles and abstracts. Among these, Seventeen articles were excluded due to missing data on primary outcome measures. Twenty review articles were excluded. Forty-one articles were excluded due to inconsistencies between the control group and experimental group. One case report and two experimental trial studies were also excluded. Ultimately, 16 articles [14–29] met the final inclusion criteria, encompassing data from 1,384 patients, and were included in the meta-analysis (Fig 1).

### 3.2 Features included in the study

Table 1 summarizes the basic characteristics of the studies included in the review. The table features Randomized Controlled Trials (RCTs) from China, India, the USA, and the UK, as well as retrospective studies from China and Italy. The sample sizes in these studies vary significantly, ranging from 40 to 200 participants, primarily targeting elderly individuals aged between 46.5 and 73.3 years. The studies employed various methods of PRP and HA injection, including simultaneous and sequential administration, with PRP doses ranging from 2 ml to 8 ml and HA doses from 0.2 ml to 3 ml. The Follow-up periods varied from 1 month to 24 months, reflecting different approaches for assessing the efficacy and safety of PRP and HA treatments.

**Table 1:** Basic Characteristics of Included Studies.

Author/Year	Country	Gender (Male/Female)	No.of patients (PRP+HA/PRP)	Age (PRP+HA/PRP)	Injection Method	Injection Dose	Injection Frequency	Follow-Up (months)
Zhao/2018 [14]	China	72/52	124 (62/62)	55.73 ± 7.18/ 56.32 ± 8.13	PRP and HA administered together	4 ml PRP + 2.5 ml HA	Once a week	1
Rao/2020 [15]	India	–	40 (20/20)	73.3 ± 7.2/ 73.3 ± 7.2	PRP administered first, HA later	4 ml PRP + 2 ml HA	Once a week	1
Ke/2016 [16]	China	49/51	100 (50/50)	57.80 ± 6.90/ 53.9 ± 7.1	PRP first, HA 10 min later	4 ml PRP + 2 ml HA	Once a week	12
Ding/2017 [17]	China	10/37	47 (20/27)	56.75 ± 9.536/ 62.11 ± 12.50	PRP and HA administered together	4 ml PRP + 2.5 ml HA	Once a week	6
Yu/2018 [18]	China	100/100	200 (104/96)	46.50 ± 7.50/ 46.20 ± 8.60	PRP and HA administered together	8 ml PRP + 0.2 ml HA	Once a week	12
Jacob/2017 [19]	USA	–	51 (20/31)	–	PRP and HA administered together	2 ml PRP	Once a week	6
Sun/2021 [20]	China	38/40	78 (39/39)	60.6 ± 8.4/ 58.4 ± 8.1	PRP and HA administered together	3 ml PRP + 3 ml HA	Once a week	6
Lana/2016 [21]	UK	13/56	69 (33/36)	62 ± 6.1/ 60.9 ± 7	PRP first, HA 10 min later	5 ml PRP + 2 ml HA	Once a week	12
Xu/2021 [22]	China	–	88 (48/40)	57.9 ± 4.1/ 56.9 ± 4.2	PRP and HA administered together	4 ml PRP + 2 ml HA	3 injections, 0.5-month intervals	24
Huang/2019 [23]	China	16/48	64 (31/33)	63 ± 7.02/ 65.03 ± 7.10	PRP and HA administered together	6 ml PRP + 2 ml HA	Once every two weeks, 3 total	6
Guo/2016 [24]	China	96/30	126 (63/63)	61.2 ± 9.6/ 60.7 ± 10.1	PRP and HA administered together	–	Once a week	12
Abate/2015 [25]	Italy	52/28	80 (40/40)	56.7 ± 11.2/ 60.90 ± 9.0	PRP and HA administered together	2 ml PRP + 2 ml HA	Once a week	6
Palco/2021 [26]	Italy	24/27	51 (28/23)	62.71 ± 7.88/ 54.04 ± 10.4	PRP + HA, centrifuged 5 min at 3400 RPM	3 ml PRP + 2 ml HA	3 injections in 30 days	12
Guo/2018 [27]	China	96/30	126 (63/63)	61 ± 10/ 61 ± 10	PRP and HA administered together	–	Once a week	12
Huang/2022 [28]	China	34/61	95 (48/47)	61.9 ± 8.8/ 61.0 ± 8.1	PRP and HA administered together	6 ml PRP + 2 ml HA	Once a week	6
Wu/2022 [29]	China	9/36	45 (22/23)	62.2 ± 1.5/ 61.3 ± 1.4	PRP first, HA 1 week later	4 ml PRP + 3 ml HA	Once a week	12

Note: PRP: Platelet-Rich Plasma; HA: Hyaluronic Acid.

### 3.3 Quality evaluation of the included literature

**Fig 2:**
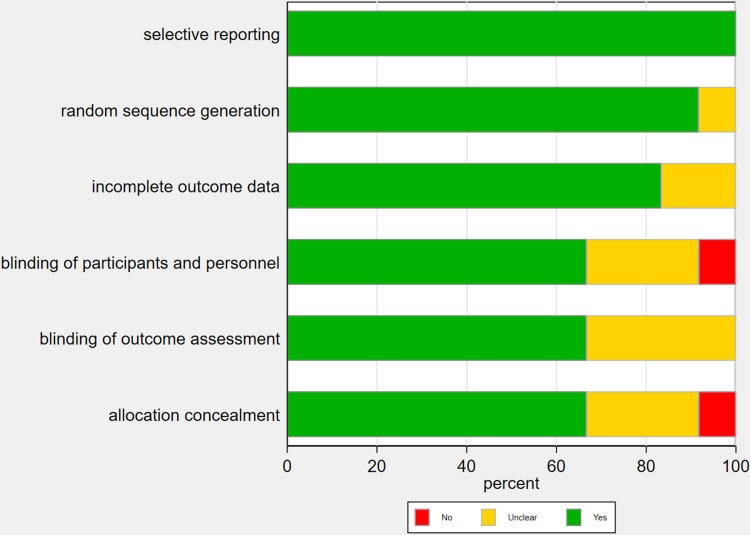
Risk of bias assessment.

**Fig 3:**
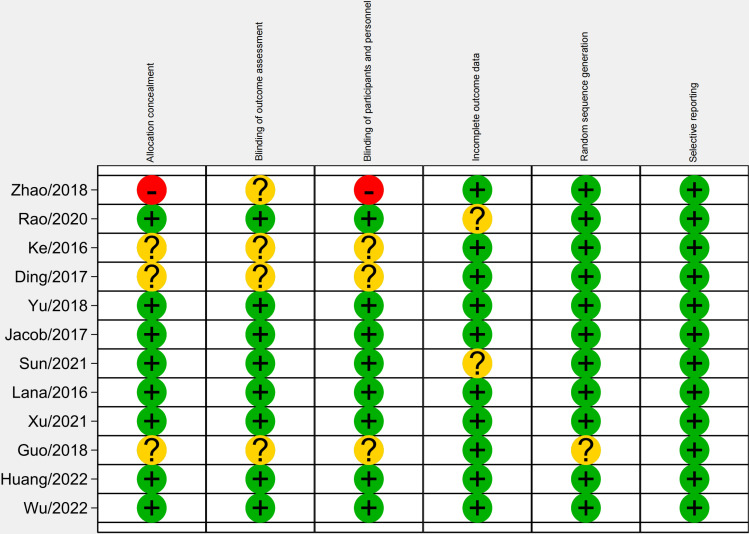
Risk of bias summary.

The risk of bias results for the randomized controlled trials indicate that most studies show low risk in areas such as random sequence generation, blinding of participants and personnel, and selective reporting, reflecting good study quality. However, some studies exhibit high or unclear risk in allocation concealment and blinding of outcome assessment, suggesting potential biases in these areas (Figs 2 and 3).

### 3.4 VAS scores

**Fig 4:**
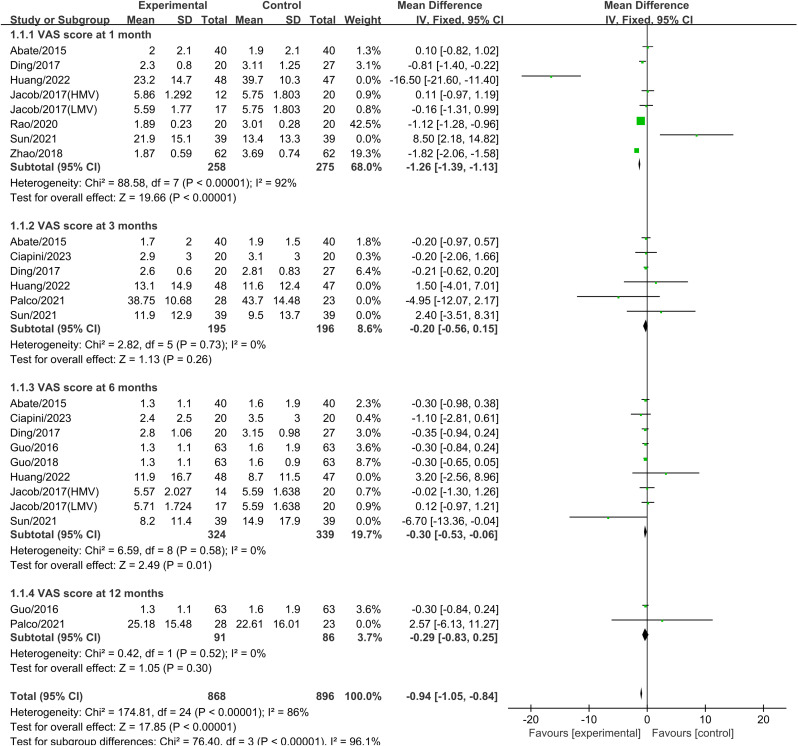
Forest plot comparing VAS scores between PRP+HA and PRP groups at 1, 3, 6, and 12 months.

Eight studies reported VAS scores one month after treatment, involving a total of 533 patients (PRP+HA group: n = 258; PRP group: n = 275). The pooled results revealed a significant difference between PRP combined with HA and PRP alone (SMD: -1.26, 95% CI: -1.39 to -1.13, P <  0.01). Heterogeneity testing revealed significant heterogeneity (I² =  97%, P <  0.001) (Fig 4).

Six studies reported VAS scores three months after treatment, involving a total of 391 patients (PRP+HA group: n = 195; PRP group: n = 196). The pooled effect size was not significantly difference between PRP combined with HA and PRP alone (SMD: -0.20, 95% CI: -0.56 to 0.15, P =  0.26) (Fig 4). Heterogeneity testing indicated no significant heterogeneity.

Nine studies reported VAS scores six months after treatment, involving a total of 663 patients (PRP+HA group: n = 324; PRP group: n = 339). Heterogeneity testing showed no significant heterogeneity (I² =  0%, P =  0.58), and a fixed-effect model was used for meta-analysis. The results indicated a statistically significant difference between PRP combined with HA and PRP alone (SMD: -0.30, 95% CI: -0.53 to -0.06, P =  0.01). The results showed that, six months post-treatment, the average VAS score in the PRP+HA group was 0.30 points lower compared to the PRP alone group (Fig 4).

Two studies reported VAS scores twelve months after treatment, involving a total of 177 patients (PRP+HA group: n = 91; PRP group: n = 86). Heterogeneity testing indicated no significant heterogeneity (I² =  0%, P =  0.52), and the results showed no significant difference between PRP combined with HA and PRP alone (SMD: -0.29, 95% CI: -0.83 to 0.25, P =  0.30) (Fig 4).

### 3.5 WOMAC total scores

**Fig 5:**
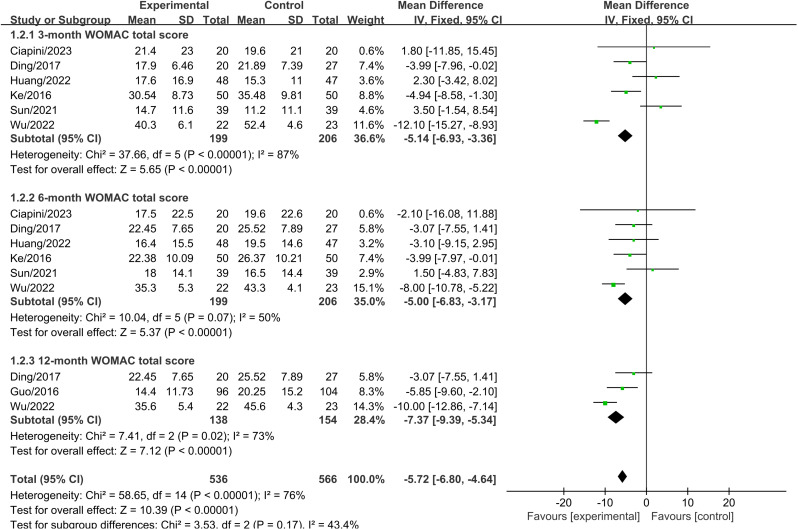
Forest plot comparing WOMAC total scores between PRP+HA and PRP groups at 3, 6, and 12 months.

Six studies, involving a total of 406 patients (PRP+HA group: n = 199; PRP group: n = 206), assessed WOMAC total scores three months after treatment. The analysis indicated no significant difference in WOMAC total scores between the experimental group and the control group at three months post-treatment (MD =  -2.87, 95% CI: -8.16 to 2.42, P =  0.29). Heterogeneity testing revealed significant heterogeneity, and a random-effects model was used (Fig 5).

Six studies, involving a total of 406 patients (PRP+HA group: n = 199; PRP group: n = 206), assessed WOMAC total scores six months after treatment. The analysis showed a significant reduction in WOMAC total scores in the experimental group compared to the control group at six months post-treatment (MD =  -3.97, 95% CI: -6.88 to -1.07, P =  0.007). This difference was statistically significant. Heterogeneity testing revealed significant heterogeneity, and a random-effects model was used (Fig 5).

Three studies, involving a total of 292 patients (PRP+HA group: n = 138; PRP group: n = 154), assessed WOMAC total scores twelve months after treatment. The analysis showed a significant reduction in WOMAC total scores in the experimental group compared to the control group at twelve months post-treatment (MD =  -6.58, 95% CI: -10.65 to -2.52, P <  0.001). This difference was statistically significant. Heterogeneity testing revealed significant heterogeneity, and a random-effects model was used (Fig 5).

**Fig 6:**
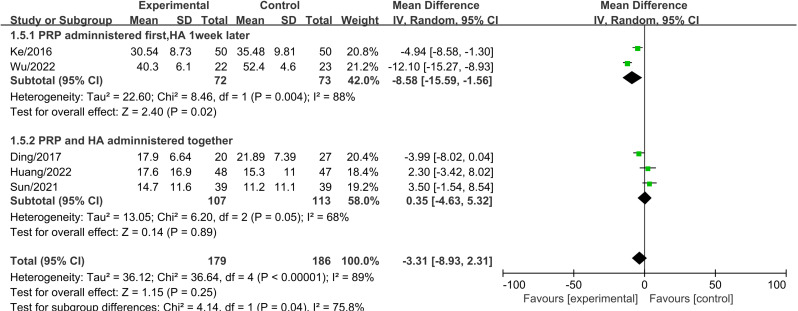
Forest plot of subgroup analysis comparing WOMAC total scores between different injection methods at three months.

A subgroup analysis was conducted based on the injection method to evaluate WOMAC total scores three months after treatment. The results indicated that administering PRP first, followed by HA one week later, led to a significant reduction in WOMAC total scores compared to the control group (MD =  -8.58, 95% CI: -15.59 to -1.56, P =  0.02) (Fig 6). This difference was statistically significant. Heterogeneity testing revealed significant heterogeneity, and a random-effects model was used. In contrast, when PRP and HA were administered together, the difference in WOMAC total scores between the two groups was not statistically significant.

**Fig 7:**
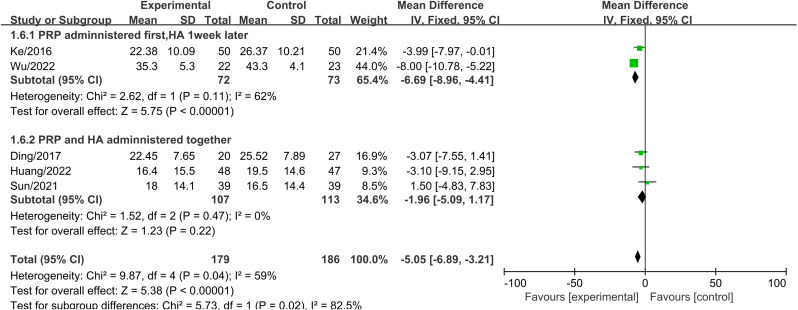
Forest plot of subgroup analysis comparing WOMAC total scores between different injection methods at six months.

A subgroup analysis was conducted based on the injection method to evaluate WOMAC total scores six months after treatment. The results showed that administering PRP first, followed by HA one week later, led to a significant reduction in WOMAC total scores compared to the control group (MD =  -6.69, 95% CI: -8.96 to -4.41, P <  0.001) (Fig 7). This difference was statistically significant. In contrast, when PRP and HA were administered together, the difference in WOMAC total scores between the two groups was not statistically significant.

### 3.6 Lequesne scores

**Fig 8:**
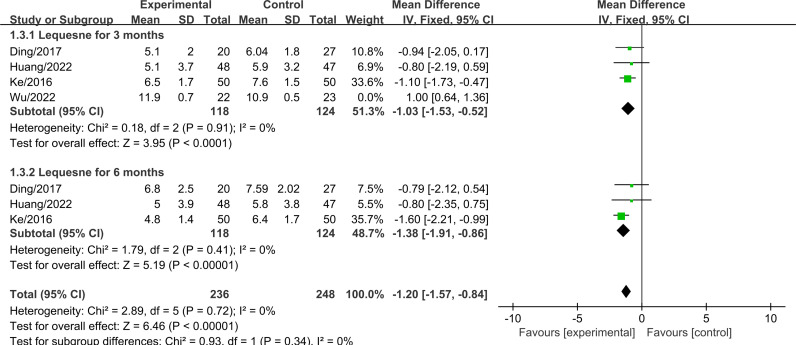
Forest plot comparing Lequesne scores between PRP+HA and PRP groups.

Four studies, involving a total of 287 patients (PRP+HA group: n = 140; PRP group: n = 147), assessed Lequesne index scores three months after treatment. Heterogeneity testing indicated significant heterogeneity (I² =  93%, P <  0.001) (S1 Fig). Sensitivity analysis was conducted by excluding studies one by one, which revealed that after excluding Wu/2022, heterogeneity decreased from 93% to 0%. The pooled analysis showed that the Lequesne index score in the experimental group (PRP+HA treatment) was significantly lower than that in the control group (PRP alone) (MD =  -1.03, 95% CI: -1.53 to -0.52, P <  0.001) (Fig 8). This difference was statistically significant.

Three studies, involving a total of 242 patients (PRP+HA group: n = 118; PRP group: n = 124), assessed Lequesne index scores six months after treatment. The analysis indicated that the Lequesne index score in the experimental group (PRP+HA treatment) was significantly lower than that in the control group (PRP alone) (MD =  -1.38, 95% CI: -1.91 to -0.86, P <  0.001) (Fig 8). This difference was statistically significant.

**Fig 9:**
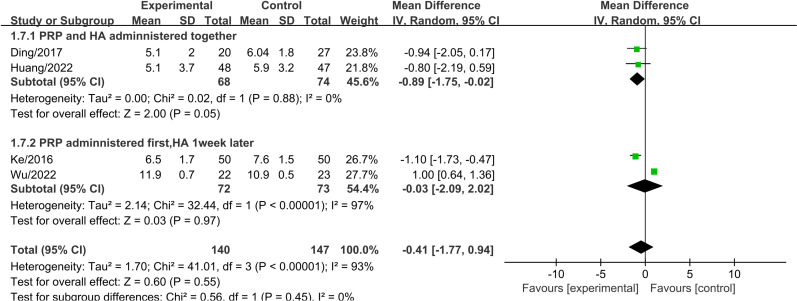
Forest plot of subgroup analysis comparing Lequesne scores between different injection methods at three months.

A subgroup analysis was conducted based on the injection method to evaluate Lequesne scores three months after treatment. The results indicated that when PRP and HA were administered together, there was a significant reduction in Lequesne scores compared to the control group (MD =  -0.89, 95% CI: -1.75 to -0.02, P =  0.05) (Fig 9). This difference was statistically significant. In contrast, when PRP was administered first followed by HA one week later, the difference in Lequesne scores between the two groups was not statistically significant.

### 3.7 Adverse events

**Fig 10:**
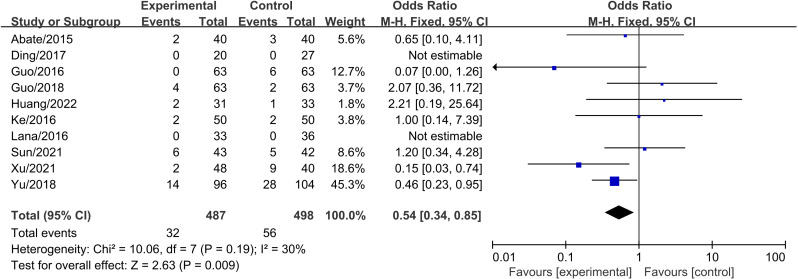
Forest plot comparing Adverse Events between PRP+HA and PRP groups.

A total of 10 studies compared PRP combined with HA to PRP alone for KOA, involving 985 patients (PRP+HA group: n = 487; PRP group: n = 498). Heterogeneity testing indicated good homogeneity (I² =  30%, P =  0.19), and a fixed-effects model was used for meta-analysis. The results showed that PRP combined with HA was associated with a lower risk of adverse events compared to PRP alone (OR =  0.54, 95% CI: 0.33 to 0.85, P =  0.009) (Fig 10).

### 3.8 Publication bias assessment

**Fig 11:**
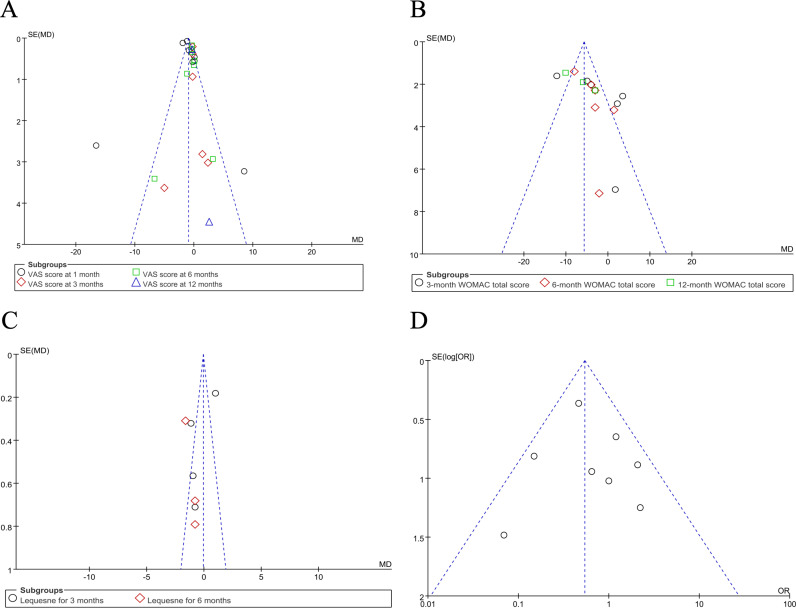
Assessment of reporting bias. A: VAS scores B: WOMAC total scores C: Lequesne scores D: Adverse Events.

The effects of VAS scores, WOMAC total scores, Lequesne scores, and Adverse Events, along with their corresponding 95% CIs, were aggregated and analyzed, and a funnel plot test was conducted to assess publication bias (Fig 11A-D). The funnel plots for VAS scores, WOMAC total scores, Lequesne scores, and Adverse Events exhibited a symmetrical distribution, indicating no evidence of publication bias.

## 4 Discussion

KOA is a common chronic joint disease characterized by degenerative changes in the knee joint cartilage and damage to the surrounding structures [30]. Approximately 20%-30% of elderly individuals are affected by KOA [31], which Chronic knee pain being the most common symptom, and in severe cases, it can impair daily activities and mobility [32], leading to altered gait and limited functionality. Treatment options for KOA are diverse, and include both nonsurgical and surgical approaches. Intra-articular injections of high-concentration PRP aim to promote tissue repair and reduce inflammation through the release of growth factors [33]. In contrast, sodium HA injections are used to lubricate the joint, reduce friction, and improve joint function [34].

Research shows that PRP promotes tissue repair by releasing growth factors such as platelet-derived growth factor (PDGF) and transforming growth factor-beta (TGF-β) [35–37]. stimulating the proliferation and regenertation of chondrocytes [38]. HA enhances the viscosity and lubricating properties of joint fluid, contributing to pain relief and improved joint function, especially in moderate to severe cases [39,40]. This meta-analysis aims to compare the efficacy and safety of PRP combined with HA versus PRP alone for the treatment of KOA.

The results from the VAS scores indicate that, in the short term, there is no statistically significant difference between PRP+HA and PRP alone, suggesting that the initial effects may be comparable. However, at six months post-treatment, intra-articular injection of PRP combined with HA was found to alleviate pain more effectively in KOA patients compared to PRP alone. Compared with PRP alone, the combination of PRP+HA significantly reduced the VAS score. The combined treatment has been shown to be more effective in relieving pain in KOA patients in the mid-term. This finding is consistent with existing research, particularly studies indicating that the lubricating effect of HA may gradually emerge after injection, thereby supporting the reparative effect of PRP over a longer period [41]. When follow-up period was extended to twelve months, the number of studies was too limited to establish statistical significance, but there was a trend toward lower VAS scores with PRP+HA compared to PRP alone [42].

The analysis of functional improvement shows, At the three-month follow-up, there was no significant difference in WOMAC total scores between PRP+HA and PRP alone (MD =  -2.87, 95% CI: -8.16 to 2.42, P =  0.29). However, at six months, the WOMAC total scores in the PRP+HA group were significantly lower than those in the PRP group (MD =  -3.97, 95% CI: -6.88 to -1.07, P =  0.007), This is consistent with the trend of functional improvement. Existing studies indicate that PRP injections can improve knee function in the short term, while HA provides longer-term support through its lubricating effects [40]. At twelve months, the WOMAC total scores in the PRP+HA group remained significantly lower compared to the PRP group (MD =  -6.58, 95% CI: -10.65 to -2.52, P <  0.001), suggesting that the combined treatment has a lasting effect on functional improvement. Additionally, the Lequesne index scores at 3 and 6 months show a trend favoring the PRP+HA group over the PRP group, further demonstrating the advantage of combined therapy in medium- to long-term functional improvement. These results are consistent with previous studies, demonstrating that combination therapy shows significant advantages in improving knee function as assessed by both WOMAC total scores and Lequesne index scores [43]. When performing subgroup analyses based on different injection methods of PRP and HA, the results indicated that administering PRP first, followed by HA one week later, yielded better WOMAC scores compared to simultaneous administration of PRP and HA. WOMAC scores provide a comprehensive assessment of pain, stiffness, and functional status, covering a broader range of daily activities.

PRP, rich in growth factors such as PDGF and TGF-β, promotes the proliferation and repair of chondrocytes. By stimulating the regeneration and repair of damaged tissue, PRP may help improve joint function and reduce pain. The effects of PRP may be more pronounced in the short term, as growth factors can immediately stimulate local tissue healing and alleviate inflammation, particularly in terms of pain relief. When PRP is administered first and followed by HA, PRP may provide significant pain relief and functional improvement in the initial phase, while HA offers long-term lubrication effects in subsequent treatment. This time interval may result in superior WOMAC scores in the later stages, as the lubrication effect of HA can manifest over a longer period.

The Lequesne scores suggest that simultaneous administration of PRP and HA may help improve joint function and reduce pain. PRP and HA may interact synergistically, leading to more pronounced effects in the short term, especially in terms of pain relief and functional impairment.

However, this study has certain limitations. First, although the meta-analysis includes multiple studies, the overall number of studies and sample sizes may still be limited. Some of the included studies have relatively small sample sizes. Second, there is some heterogeneity in the meta-analysis of VAS scores, WOMAC total scores, and Lequesne scores. For example, VAS scores at different time points exhibited significant heterogeneity (e.g., I² =  97%). This heterogeneity may be attributed to differences in PRP and HA injection protocols, baseline patient characteristics, and study design variations among the studies. Third, while the study assessed outcomes at 1 month, 3 months, 6 months, and 12 months, some studies only provided data for shorter time points (e.g., 1 month). Data on long-term effects (e.g., beyond 12 months) are relatively sparse, which may limit a comprehensive evaluation of the long-term effects of combined PRP and HA treatment.

## 5 Conclusion

This study demonstrates that combined treatment with PRP and HA for KOA offers superior pain relief and functional improvement compared to PRP alone. Although both treatments show similar effectiveness in the short term (1 month post-treatment), the combined therapy significantly outperforms PRP alone in terms of long-term pain relief and functional improvement at 6 and 12 months, particularly in WOMAC total scores and Lequesne index scores. Furthermore, the combined therapy exhibits better safety compared to PRP alone. Subgroup analysis also revealed that administering PRP followed by HA yields better outcomes than simultaneous administration, suggesting that the sequence of treatment may impact its effectiveness.

## Supplementary infomation

S1 Fig Forest plot comparing Lequesne scores between PRP+HA and PRP groups. (PNG)

S1 Table PRISMA checklist. (DOCX)

File S1 List of papers included in this meta-analysis. (XLSX)

File S2 List of papers excluded in this meta-analysis. (XLSX)

File S3 Study characteristics assessing the quality of the included studies. (XLSX)
