# Supplementary figures and images for: The efficacy and safety of intra-articular platelet-rich plasma versus sodium hyaluronate for the treatment of osteoarthritis: Meta-analysis
Source: PLoS One. 2025 Mar 21;20(3):e0314878. doi: 10.1371/journal.pone.0314878 (PMC11927909; doi:10.1371/journal.pone.0314878)

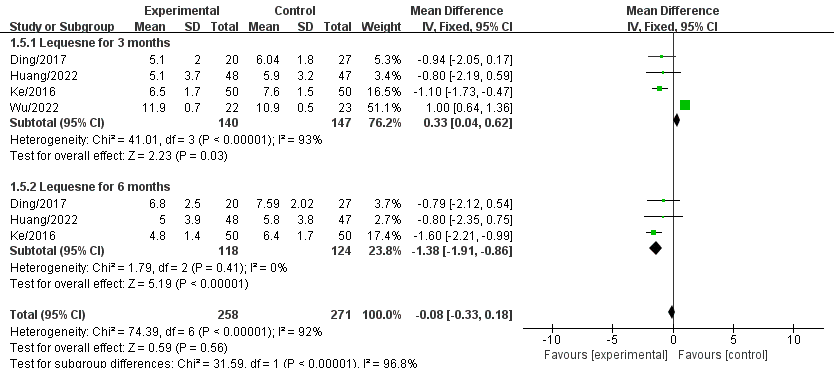

Supplement: S1 Fig — (PNG) [file pone.0314878.s001.png]
